# The *para^bss1^ Drosophila melanogaster* as Model for Chronic Nociception: Insights Into Cannabidiol Analgesic Effects

**DOI:** 10.1002/ejp.70225

**Published:** 2026-01-27

**Authors:** Serena Mares Malta, Lucas Ian Veloso Correia, Alexandre Souza Marquez, Lucas Matos Martins Bernardes, John George Howland, Ana Paula Mendes‐Silva, Foued Salmen Espíndola, Carlos Ueira‐Vieira

**Affiliations:** ^1^ Laboratory of Genetics, Institute of Biotechnology Federal University of Uberlandia Uberlandia Minas Gerais Brazil; ^2^ Department of Psychiatry University of Saskatchewan Saskatoon Saskatchewan Canada; ^3^ Laboratory of Nanobiotechnology, Institute of Biotechnology Federal University of Uberlandia Uberlandia Minas Gerais Brazil; ^4^ Department of Anatomy, Physiology and Pharmacology University of Saskatchewan Saskatoon Saskatchewan Canada; ^5^ Laboratory of Biochemistry and Molecular Biology, Institute of Biotechnology Federal University of Uberlândia Uberlandia Minas Gerais Brazil

**Keywords:** cannabidiol, chronic pain model, *Drosophila melanogaster*, nociception, *para* ^*bss1*^ mutant

## Abstract

**Background:**

Chronic pain, which is often unrelated to ongoing injury, is poorly understood and difficult to treat. Genetic studies have identified voltage‐gated sodium (Na_v_) channels, particularly gain‐of‐function mutations such as L858F and R1150W in human Na_V_1.7, as involved in the development of chronic pain.

**Methods:**

A chronic pain model was proposed in *Drosophila* using the *para* ^*bss1*^ mutant. Behavioural chemical nociceptive assay was conducted, and sensitivity was pharmacologically tested with carbamazepine and cannabidiol to assess the model's validity for analgesic screening.

**Results:**

Sequence alignment and 3D structural modelling revealed strong homology between human Na_v_1.7 and the *para* gene, though no structural alterations were observed between the parabss1 allele and the wild‐type allele. Functionally, *para* ^*bss1*^ larvae exhibited enhanced sensitivity to chemical, nociceptive stimuli compared to *w* ^*1118*^ larvae. Furthermore, carbamazepine increased response latency in *w* ^*1118*^; however, *para* ^*bss1*^ showed a time and dose‐dependent response to this treatment. Oral administration of cannabidiol significantly increased latency to chemical stimuli in both genotypes, supporting cannabidiol's modulatory role in nociceptive circuits. These findings validate the *para* ^*bss1*^ mutant as a tractable in vivo platform for chronic nociception studies and pharmacological screening.

**Conclusions:**

The *para* ^*bss1*^ mutant demonstrates heightened chemical nociception, resistance to carbamazepine and sensitivity to cannabidiol, thereby validating it as a pertinent *Drosophila* model for chronic pain. This model facilitates the screening of candidate analgesics targeting sodium channel dysfunctions in an in vivo setting, thereby demonstrating translational potential.

**Significance Statement:**

This study proposes the *Drosophila melanogaster para* ^*bss1*^ mutant as a valid and manageable in vivo model for chronic nociception. By exhibiting selective hypersensitivity, resistance to conventional treatment and sensitivity to cannabidiol, this model provides a cost‐effective and ethically favourable platform for the preclinical screening of novel analgesics that target sodium channel dysfunctions. This study opens a new avenue for translational pain research and aligns with the ongoing demand for alternative animal models in pain therapeutic development.

## 1 Introduction

Pain is a complex and multidimensional phenomenon. The International Association for the Study of Pain (IASP) defines it as “an unpleasant sensory and emotional experience associated with actual or potential tissue damage” (Treede 2018). This definition distinguishes pain, a conscious perspective, from nociception, which is the neural process that detects and transmits potentially harmful stimuli (Ang et al. 2017; Mitsi and Zachariou 2016). Nociceptors, specialized peripheral neurons, initiate this process by detecting noxious mechanical, thermal or chemical inputs, and two main fibre types are involved: thinly myelinated fibres, which convey sharp and localised pain rapidly, and unmyelinated C fibres, which transmit dull and diffuse pain more slowly (Basbaum et al. 2009; Campbell and Meyer 2006; Tracey 2017).

Chronic pain, defined as persisting beyond 3 months, affects up to 70% of older adults and often emerges independently of ongoing tissue injury (Bennett et al. 2019; Breivik et al. 2006; Patel et al. 2013). Its pathophysiology remains incompletely understood, encompassing complex interactions between peripheral and central sensitization, neuroimmune processes and individual genetic predisposition. Several genes implicated in neurotransmission and immune response are associated with chronic pain susceptibility, including those encoding ion channels such as voltage‐gated sodium (Na_v_) and calcium channels (Ca_v_), and mutations in these genes can alter nociceptor excitability, contributing to development of chronic pain through channelopathies (Bamps et al. 2021; Dohrn et al. 2019; Stenz et al. 2022).

Among sodium channels, Na_v_1.7 (encoded by SCN9A) plays a central role in human pain physiology. Gain‐of‐function mutations in Na_v_1.7 are associated with inherited erythromelalgia and paroxysmal extreme pain disorder, whereas loss‐of‐function mutations result in congenital insensitivity to pain (Cox et al. 2006; Dib‐Hajj et al. 2005). Because of this dual relevance, Na_v_1.7 is considered a key molecular determinant of nociception and a major target for analgesic development.

Despite the availability of Nonsteroidal anti‐inflammatory drugs (NSAIDs), antidepressants, anticonvulsants and opioids to alleviate pain, their efficacy is limited in chronic conditions (Cohen et al. 2021; Park and Moon 2010). Carbamazepine, for instance, is prescribed for trigeminal neuralgia and other neuropathic conditions but is associated with notable adverse effects (Guo et al. 2024; Wiffen et al. 2014).

These therapeutic limitations underscore the need for alternative strategies (Mohammed et al. 2024). Cannabidiol (CBD), a non‐psychoactive phytocannabinoid, has been reported to have analgesic, anti‐inflammatory and neuroprotective properties (Mohammed et al. 2024). Preclinical and clinical studies suggest its potential, but further investigation is necessary to clarify its mechanisms and therapeutic value (Schwan et al. 2019).

To address both the need for reliable experimental models and the exploration of novel treatments, we propose a *Drosophila melanogaster* model of chronic nociception using the *para* ^*bss1*^ mutant. In flies, the *para* gene encodes the principal voltage‐gated sodium channel, functionally analogous to vertebrate Na_v_ channels. Two sodium‐channel genes exist in the fly genome: Na_v_1, the predominant neuronal isoform and Na_v_2, which remains poorly characterised (Liebeskind et al. 2011; Zakon 2012). The *para* ^*bss1*^ mutant (L1699F) carries a gain‐of‐function substitution that enhances neuronal excitability, lowers the seizure threshold and leads to the classical bang‐sensitive phenotype in adults (Kroll et al. 2015; Loughney et al. 1989; Parker et al. 2011). This mutation also leads to hypersensitivity and resistance to classical Na^+^ channel blockers such as carbamazepine (Ganetzky 2000; Kroll et al. 2015; Parker et al. 2011). Electrophysiological recordings from *Xenopus oocytes* expressing the mutant channel confirmed altered inactivation dynamics consistent with sodium‐channel hyperactivity (Parker et al. 2011). Due to the evolutionary conservation of sodium channels between insects and mammals, this model represents a promising platform for screening compounds that modulate nociceptive pathways, especially those targeting persistent pain states.

In this study, we aim to establish a model of chronic nociception by integrating behavioural, pharmacological and comparative structural analyses. We evaluate whether the gain of function mutation in the para channel enhances nociceptive sensitivity and whether pharmacological agents such as carbamazepine and cannabidiol modulate this response. Moreover, through sequence and 3D structure analyses, we compare *Drosophila para* with human Na_v_1.7 to assess their evolutionary and translational relevance. As proof of concept, we also assess the effects of CBD, providing initial insights into potential analgesic. While the primary focus is the proposal of an in vivo platform for studying nociception, the inclusion of CBD tests may contribute to expanding pharmacological perspectives for pain management.

## 2 Methods

### 2.1 Flies Stock

The *para* ^*bss1*^ *D. melanogaster* strain was kindly provided by Dr. Tanouye (University of California, Berkeley). The *w* ^*1118*^ (BL #3605) was obtained from the Bloomington Drosophila Stock Center. The flies were fed with standard cornmeal food (Bloomington) *ad libitum* and kept at 25°C on a 12‐h dark/light cycle.

### 2.2 Comparison of Human and Fly Sodium Channels Using BLASTp

To explore the evolutionary relationship and structural conservation between human and *Drosophila melanogaster* sodium channels, particularly those involved in pain perception, BLASTp alignments were performed to identify potential homologous regions. The human proteins analysed were SCN9A (Na_v_1.7) (access number NP_002968.1), SCN10A (Na_v_1.8) (access number NP_001280235.2) and SCN11A (Na_v_1.9) (access number NP_001336182.1), all from the current genome version GRCh38.p13.

The protein encoded by the *para* gene from *Drosophila melanogaster* (access number NP_001188627.1) current genome version 6.32 was aligned against human protein using Protein BLAST: Align two or more sequences using BLAST.

### 2.3 Prediction of 3D Proteins Structures and Alignments

To investigate whether specific gain‐of‐function mutations in human and *Drosophila melanogaster* sodium channels result in structural changes that could explain variations in channel activity, we performed comparative protein modelling and structural alignment. The 3D structure models were predicted using Swiss‐Model (https://swissmodel.expasy.org). Following the upload of protein sequences, the program conducted a search for 3D templates. The best templates were used for building 3D models. UCSF Chimera was used to visualise and analyse the molecular structures (Pettersen et al. 2004). The RaptorX was used for alignment of predicted 3D structures (Wang et al. 2011, 2013) and assess potential conformational differences between the wild‐type and mutant channels.

### 2.4 Chemical Nociception Test

The nociception test was adapted from Lopez‐Bellido et al. (2019). Solutions of HCl were generated ranging from 0.5% to 9% (vol/vol) from HCl stock (Sigma: 37%) diluted in Milli‐Q water. Third instar larvae (*n* ≥ 15) were collected and placed in a glass petri dish and allowed to explore for approximately 10 s. They were then exposed to a given solution by pipetting 1.5 μL onto the posterior end of the larvae. Larvae behavior was recorded and analysed using QuickTime Player 10.5 version. A complete roll of 360° along the body axis within 10 s of exposure was scored as an aversive behavior. Other characteristic behaviors were not categorised.

### 2.5 Carbamazepine Treatment

Third instar larvae were exposed to a preparation of instant mashed potato (Yoki) hydrated with 2 mL solution of Carbamazepine at concentrations of 3 or 6 mg/mL.

Carbamazepine (300 mg) was first dissolved in 1 mL of absolute ethanol then diluted in distilled H_2_O to 3 or 6 mg/mL. Two mL of each solution at the final concentration were mixed with 2 mg of instant mashed potato (Yoki) and placed in a petri dish. The control group was fed with a preparation containing only water, while the treatment group received the Carbamazepine‐enriched solution; third instar larvae were added in the center of the preparations and kept at 25°C. The larvae were then tested for chemical nociception using 2% HCl as the noxious stimulus (*n* ≥ 15 per group) after 1 and 6 h of exposure to the treatment.

### 2.6 Cannabidiol (CBD) Treatment

Third instar larvae were collected and fed a hydrated instant mashed potato medium (Yoki) supplemented with CBD (Mahara, Brazil) at a final concentration of 0.1 mg/mL, prepared in a 3% PEG400 solution (vehicle). Control larvae were fed with a solution containing only vehicle and food colouring, while the treatment group received the CBD‐enriched solution. Both groups were maintained at 25°C and allowed to feed for 1 h. After feeding, larvae were tested for chemical nociception using 2% HCl as a noxious stimulus (*n* ≥ 15 per group).

### 2.7 Statistical Analysis

The D'Agostino and Pearson test was used for normality distribution. Groups were compared by *t*‐test (parametric) or Kruskal Wallis test (non‐parametric) with an established significance level of *p* ≤ 0.05. Two‐way and one‐way analysis of variance (ANOVA) with Bonferroni post hoc correction was used for multiple group comparisons. All analyses were performed using the software GraphPad Prism 8.

## 3 Results

### 3.1 Comparation Mutation in Na_v_ Proteins Human and *Drosophila melanogaster*

To identify potential structural similarities between human pain‐related sodium channels and the Drosophila para channel, we first compared their protein sequences and modelled their structures. This analysis aimed to support the translational relevance of using para mutants to study Na_v_‐dependent nociception.

The sequence of human proteins Na_v_1.7, Na_v_1.8 and Na_v_1.9 was compared to *D. melanogaster* by BLASTp to assess structural changes in human and *D. melanogaster* Na_v_ proteins. All identified mutations described in Table 1 promote a gain of function in the protein and are associated with inherited pain syndromes or other neurological conditions. While Na_v_1.8 and Na_v_1.9 did not exhibit a conserved motif associated with *para* ^*bss1*^, Na_v_1.7 displayed the highest query coverage with the *para* gene fly (95%). Therefore, Na_v_1.7 and *para* ^*bss1*^ were selected for the prediction of 3D protein structures in *D. melanogaster* .

**TABLE 1:** Comparative analysis of voltage‐gated sodium channels (Nav) in *Homo sapiens* and *Drosophila melanogaster* .

Organism	Sodium Channel	Gene	Mutation	Blast P against *para D. melanogaster*	
				Query cover	Identity
*Homo sapiens*	Na_v_1.7	SCN9A	Q10R I136V S211P F216S I234T S241T I245V N395K V400M G616R L823R L848T G856D L858H L858F A863P V872G Q875E L955Del P1308L V1316A F14449V W1538R A1632T A1746G	95%	43.85%
	Na_v_1.8	SCN10A	L554P M650K A1304T G1662S I1706V	78%	47.83%
	Na_v_1.9	*SCN11A*	R222H R222S R225C I381T G699R A808G L811P L1158P N1169S V1184A I1293V L1302F	80%	43.92%
*Drosophila melanogaster*	Na_v_1.1	*para*	L1699F or L1678F	100%	100%

*Note:* Human genes *SCN9A*, *SCN10A*, and *v* encode Na_v_1.7, Na_v_1.8, and Na_v_1.9 channels, respectively, all implicated in nociception and chronic pain. The *Drosophila* homologue is the *para* gene, encoding the para sodium channel. Listed mutations represent known pathogenic or functionally relevant variants. BLASTp alignment scores indicate the degree of sequence similarity between each human channel and the *Drosophila* para channel, expressed as query cover and identity percentage. The *para* ^*bss1*^ mutation (L1699F or L1678F) is also noted.

**FIGURE 1:**
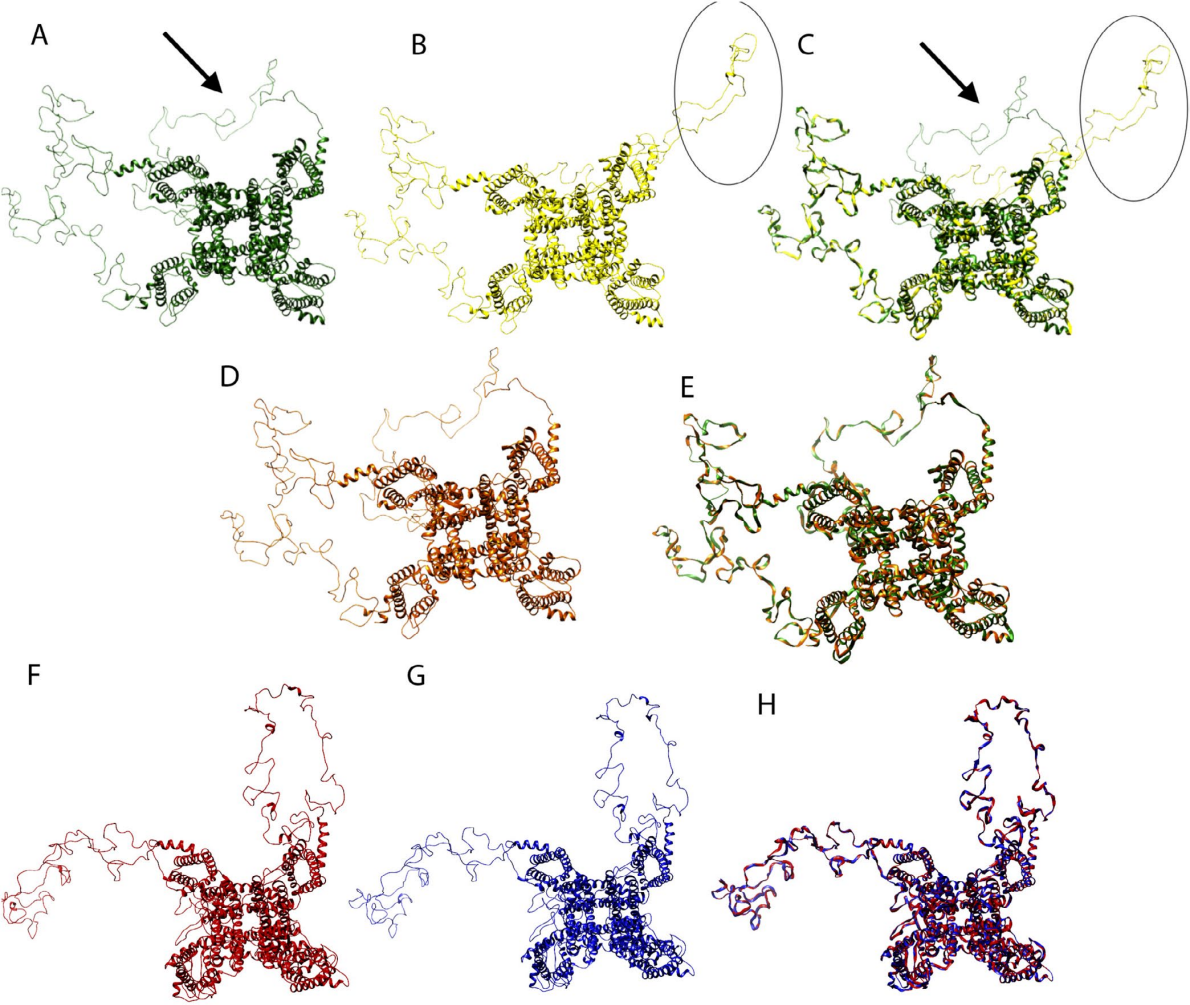
Predicted 3D models for Sodium channel. (A) Na_V_ 1.7 human wild type. (B) mutant Na_V_ 1.7 human (R1150W). (C) overlay between e Na_V_ 1.7 wild type (green) and mutant (R1150W) (yellow). (D) mutant NaV 1.7 human (L858F). (E) overlay between and Na_V_ 1.7 wild type (green) and mutant (L858F) (orange). (F) 3D models of wild type *para* ^*bss1*^ *Drosophila melanogaster* sodium channel. (G) model for *para* ^*bss1*^ mutant of *D. melanogaster* (L1678F). Arrow and circles in A–C shows conformational differences.

The Na_v_1.7 had 78.51% identity with the human sodium channel protein type 2 subunit alpha PDB 6j8e.1.B. These templates were used to predict the 3D model of *para* and *para* ^*bss1*^ for *D. melanogaster* and Na_v_1.7 wild type, as well as two distinct models for the mutation R1150W and L858F. The analysis of the overlap among human 3D structures showed that the R1150W mutation promotes a conformational change in a cytoplasmatic domain (Figure 1A–C).

Specifically, our analysis indicates that this change involves a structural rearrangement in cytoplasmic loops, which span residues mainly SER490–SER695 and SER1004–THR1145. The R1150W mutation, located adjacent to this region, appears to alter the spatial orientation of the C‐terminal portion of this loop, leading to its more exposed conformation in the mutant channel. To support these structural comparisons, we calculated the Root‐Mean‐Square Deviation (RMSD) between the models (Table 2). This analysis confirms that while the overall fold between human Na_v_1.7 and *Drosophila para* is conserved, the L1699F mutation in para does not induce a major structural alteration (RMSD = 0.0 Å), unlike the human R1150W mutation.

**TABLE 2:** Descriptive comparison between human Na_v_1.7 and Drosophila para channel structures.

Structure alignment	Root‐mean‐square deviation (RMSD)
Human Na_V_ 1.7 × para wild type	4.57
Human Na_V_ 1.7 × para mutant bss	4.49
para Wild Type × para mutant bss	0.0
Human Na_V_ 1.7 × para wild type × para mutant bss	3.91

*Note:* RMSD values were calculated to assess the structural similarity between the human Na_v_1.7 channel and *Drosophila* para channels (wild type and mutant *para* ^*bss1*^). Lower RMSD values indicate higher structural similarity. The para wild type and mutant *para* ^*bss1*^ structures showed complete overlap (RMSD = 0.0), while both displayed moderate deviations when compared to human Na_v_1.7.

In contrast, the L858F mutation did not promote any conformational changes (Figure 1A,G,E). For *D. melanogaster*, the overlap of predicted 3D structures of the proteins from *para* (wild‐type) and *para* ^*bss1*^ showed that there are no structural differences between them.

### 3.2 Validation of Chemical Nociception in *D. melanogaster* *w* ^*1118*^

Before testing mutant strains, we confirmed a reliable chemical nociception assay in reference strain *w* ^*1118*^. This step aimed to define stimulus conditions that evoke nocifensive rolling responses.

**FIGURE 2:**
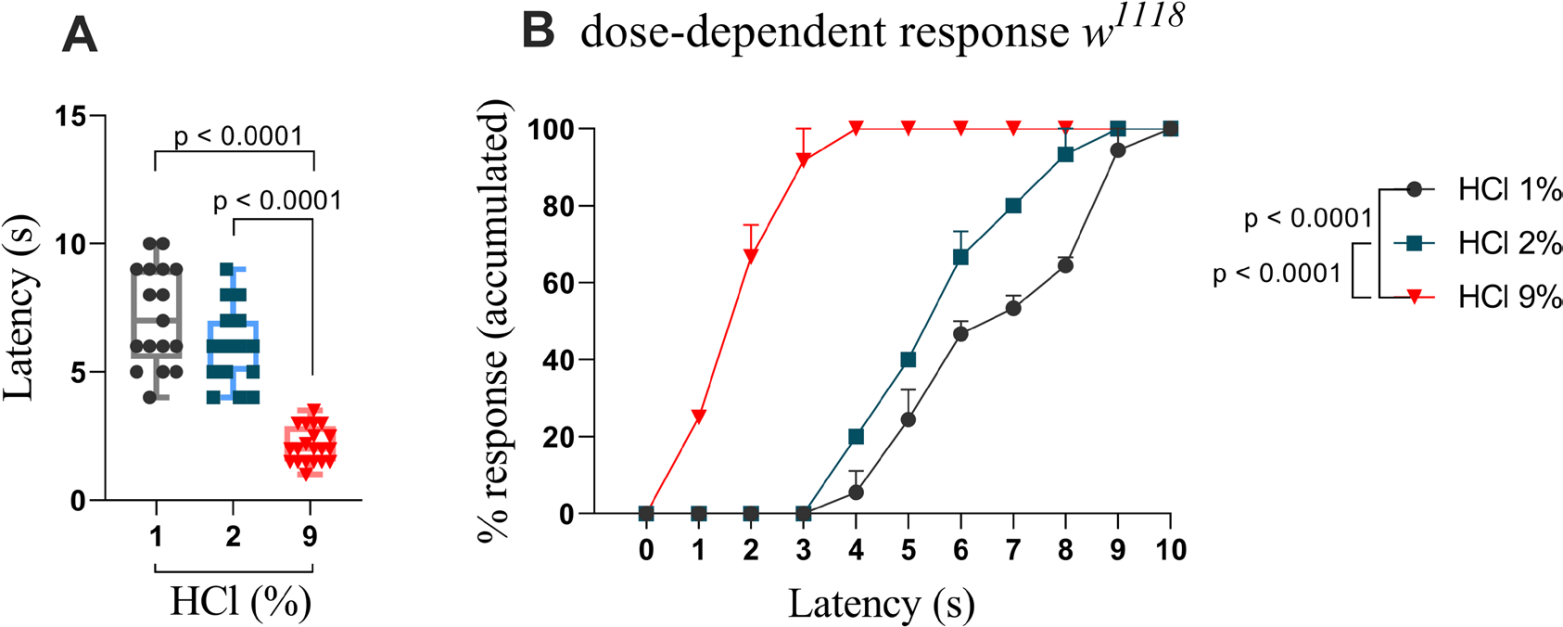
Validation of a chemical nociception assay using different concentrations of hydrochloric acid in *Drosophila melanogaster* larvae. (A) *w* ^*1118*^ larvae treated with 9% HCl showed significantly increased responsiveness compared to those treated with 2% or 1% HCl (*p* < 0.0001). No significant difference was observed between the 1% and 2% HCl groups (*p* = 0.0798). One‐way ANOVA test was performed. (B) Accumulated dose‐dependent HCl response. Boxes represent the interquartile range, with the horizontal line indicating the mean. The whiskers represent the minimum and maximum values, respectively. Data are presented as individual data points for each group (*n* ≥ 15 larvae per group).

Our results showed that third instar larvae are very sensitive to treatment with 9% HCl compared to 2% and 1% HCl (*p* < 0.0001), while no difference was found between treatment with 2% and 1% HCl (*p* = 0.0798) (Figure 2). One hundred percent of larvae responded within 3 s, whereas only 50% responded by 6 s following treatment with 2% and 1% of HCl.

### 3.3 *para^bss1^* is More Sensitive to Chemical Nociception Than *w* ^*1118*^

To determine whether the gain‐of‐function mutation in the para gene affects chemical nociception, we next compared the behavioural responses of *w* ^*1118*^ and *para* ^*bss1*^ larvae to noxious HCl. This experiment tested the hypothesis that increased sodium channel excitability enhances sensitivity to chemical stimuli.

**FIGURE 3:**
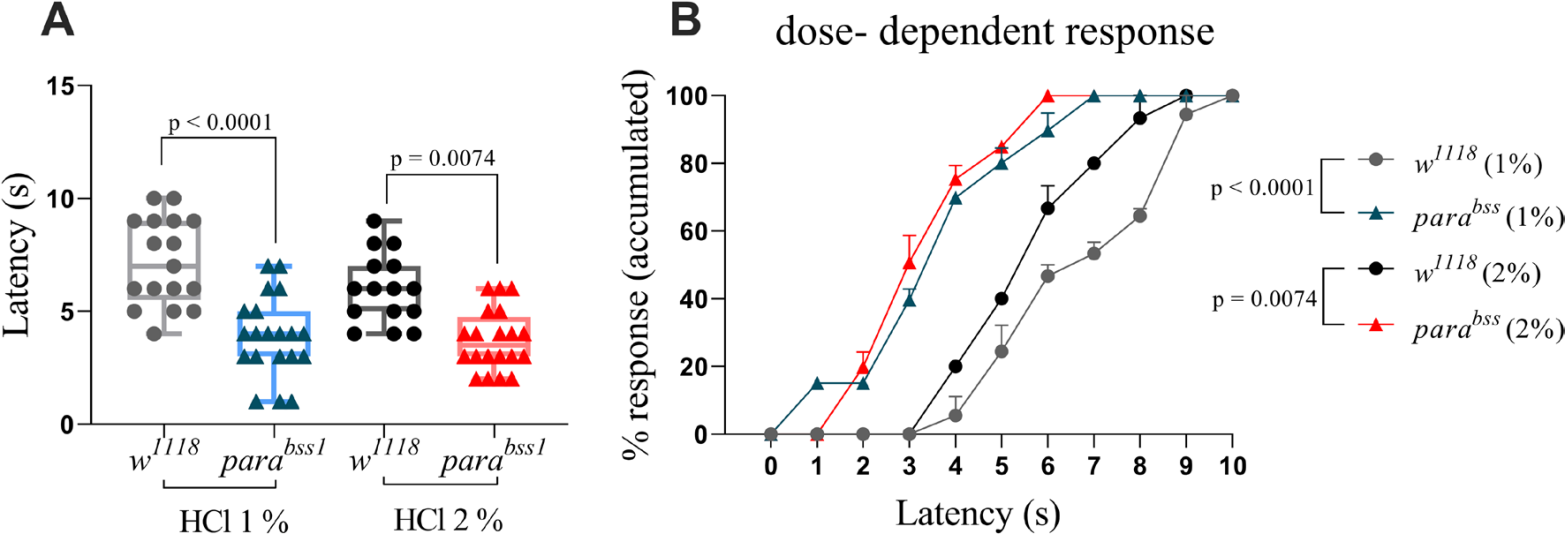
*para* ^*bss1*^ exhibit increased sensitivity to chemical nociception compared to *w* ^*1118*^. (A) Mean response times to 1% and 2% HCl were significantly shorter in third‐instar *para* ^*bss1*^ larvae compared to *w* ^*1118*^. Statistical analysis using two‐way ANOVA revealed *p* < 0.0001 for 1% HCl and *p* = 0.0008 for 2% HCl. (B) Cumulative percentage of larvae displaying a full 360° nociceptive rolling response over time. When exposed to 1% HCl, 60% of *para* ^*bss1*^ larvae responded within 3 s, whereas *w* ^*118*^ larvae showed no response at this time point (*p* < 0.0001). Under 2% HCl exposure, 60% of the *para* ^*bss1*^ responded within 4 s, while *w* ^*1118*^ larvae only reached that threshold by 6.5 s (*p* = 0.0008). Each box represents the interquartile range, with the horizontal line indicating the mean. The whiskers represent the minimum and maximum values, respectively. Data are presented as individual data points (*n* ≥ 15 larvae per group).

The *para* ^*bss1*^ mutant third instar larvae, when exposed to the noxious chemical stimulus, had a response time below 5 s and significantly lower than *w* ^*1118*^ flies (*p* < 0.0001 for 1% HCl and *p* = 0.0008 for 2% HCl) (Figure 3A).

The accumulated response in percentage analyses (Figure 3B) showed that 60% of the *para* ^*bss1*^ third instar larvae tested showed completed a 360° roll in 3 s, while none of the *w* ^*1118*^ larvae had the same behaviour at the same time (*p <* 0.0001) when both strains were treated with 1% HCl. When exposed to 2% HCl, *para* ^*bss1*^ larvae reached 60% response at 4 s, while *w* ^*1118*^ required 6.5 s to reach the same threshold (*p* = 0.0008 one‐way ANOVA analysis). Significant differences were found through two‐way ANOVA analysis, at 5 s (*p* = 0.0026), 6, 7 and 8 s (*p* < 0.0001) when comparing only *w* ^*1118*^. When *para* ^*bss1*^ was compared across time points, no significant differences were observed (*p* > 0.05).

### 3.4 Differential Sensitivity of *para^bss1^* and *w* ^*1118*^ Larvae to Na^+^ Channel Blocker

**FIGURE 4:**
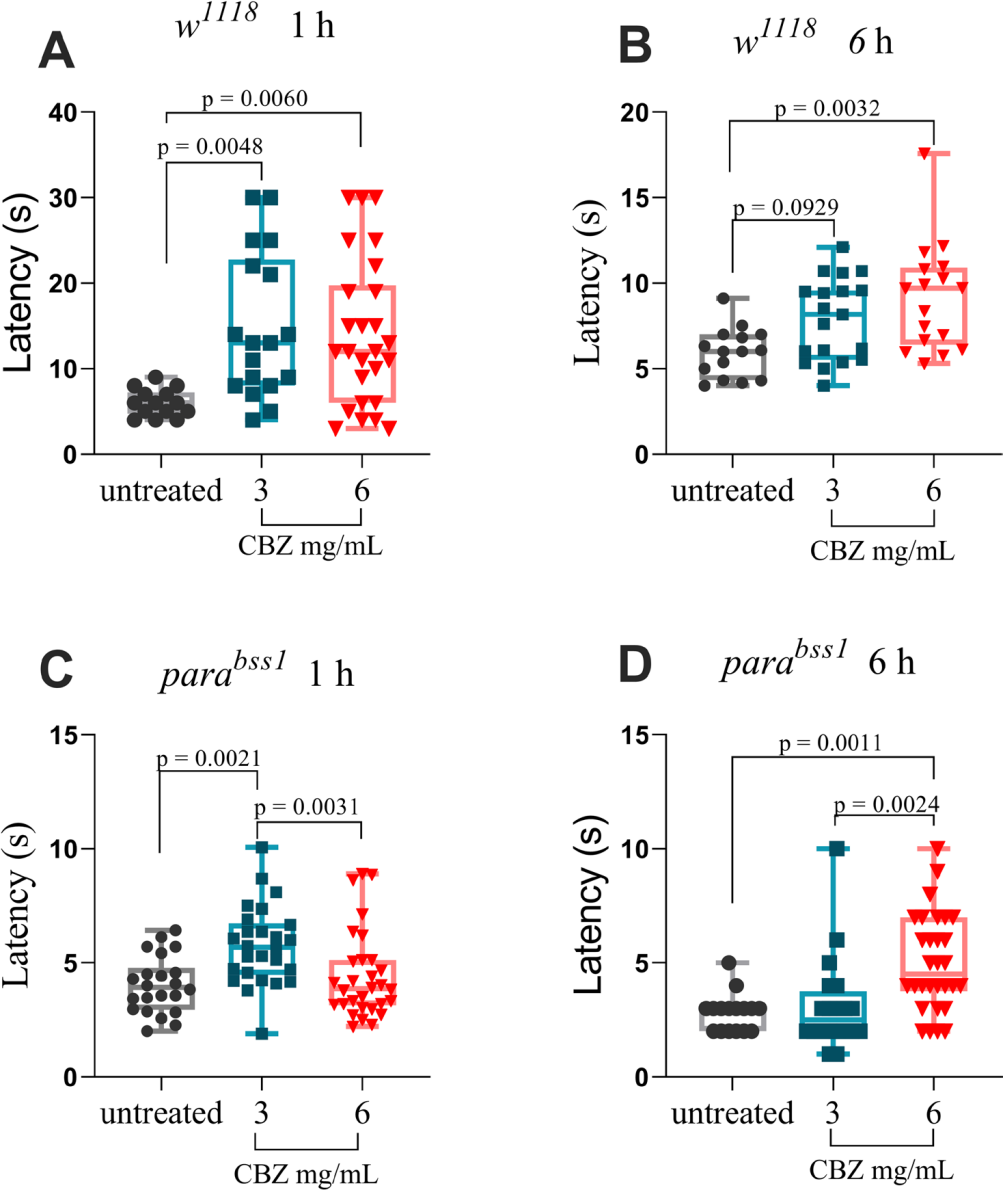
Time‐ and dose‐dependent effects of carbamazepine on chemical nociception in *w* ^*1118*^ and *para* ^*bss1*^ larvae. (A) In *w* ^*1118*^ third‐instar larvae, exposure to carbamazepine (CBZ) at concentrations of 3 mg/mL and 6 mg/mL for 1 h significantly increased nociceptive response latency compared to untreated controls (*p* = 0.0048 and *p* = 0.0060, respectively; one‐way ANOVA with Tukey's post hoc test). No significant difference was observed between concentrations (*p* > 0.9999). (B) After 6 h of exposure, only the 6 mg/mL group remained significantly different from controls (*p* = 0.0032; Kruskal‐Wallis test), while the 3 mg/mL group showed no significant change (*p* = 0.0929). (C) In para^bss1^ larvae, treatment for 1 h with 3 mg/mL CBZ significantly increased latency compared to untreated controls (*p* = 0.0021), whereas 6 mg/mL had no effect (*p* > 0.9999); latency at 3 mg/mL was higher than at 6 mg/mL (*p* = 0.0031) (Kruskal‐Wallis test). (D) After 6 h, the 6 mg/mL treatment significantly increased latency compared to both untreated (*p* = 0.0011) and 3 mg/mL groups (*p* = 0.0024) one‐way ANOVA. Each box represents the interquartile range, with the horizontal line indicating the mean; whiskers show the minimum and maximum values. Data are presented as individual data points (*n* ≥ 10 larvae per group).

To evaluate whether the *para* ^*bss1*^ mutation alters sensitivity to sodium‐channel blockade, both genotypes were treated with carbamazepine (CBZ) and subsequently challenged with 2% HCl in the chemical nociception assay (Figure 4A–D).

After 1 h of exposure (Figure 4A), *w* ^*1118*^ larvae treated with CBZ 3 mg/mL and 6 mg/mL showed an increase in latency compared with untreated controls (*p* = 0.0048) and (*p* = 0.0060), respectively; as determined by one‐way ANOVA: *F* (2, 55) with Tukey's post hoc test. The treatment groups exhibited mean latencies of 14.89 ± 8.46 s (3 mg/mL), 14.08 ± 8.60 s (6 mg/mL) and 6.00 ± 1.62 s (untreated), with no significant difference between concentrations of CBZ (*p* > 0.9999). When exposure was extended to 6 h (Figure 4B), *w* ^*1118*^ larvae treated with 6 mg/mL of CBZ showed a significant increase in latency compared with the untreated group (*p* = 0.0032) while no significant difference was detected between untreated and 3 mg/mL group (*p* = 0.0929), or between the two CBZ concentrations (*p* = 0.6473) Kruskal‐Wallis H(2) = 9.305 with Dunn's post hoc test.

In contrast, *para* ^*bss1*^ larvae exhibit a distinct temporal profile. After 1 h of CBZ exposure (Figure 4C) the 3 mg/mL group showed a significant increase in latency compared with untreated controls (*p* = 0.0021), whereas 6 mg/mL did not differ from untreated (*p* > 0.9999) Kruskal‐Wallis H(2) = 11.37 and Dunn's post hockey test. Mean latencies were 4.42 ± 2.15 s (untreated), 5.58 ± 2.34 s (3 mg/mL) and 4.73 ± 1.92 s (6 mg/mL). However, after 6 h of exposure (Figure 4D), *para* ^*bss1*^ larvae treated with 6 mg/mL CBZ exhibited a significant increase in latency compared with both untreated and 3 mg/mL‐treated groups (*p* = 0.0011 and *p* = 0.0024, respectively), and no difference was detected between untreated and 3 mg/mL groups (*p* > 0.9999) according to one‐way ANOVA, *F*(2,63) = 6.92 and Bonferroni's post hockey test. The mean latencies were 2.81 ± 0.83 s (untreated), 3.10 ± 2.05 s (3 mg/mL) and 5.50 ± 2.13 s (6 mg/mL).

### 3.5 Cannabidiol (CBD) Significantly Increased Chemical Nociception Latency in *w^1118^* and *para* ^*bss1*^ Mutant

**FIGURE 5:**
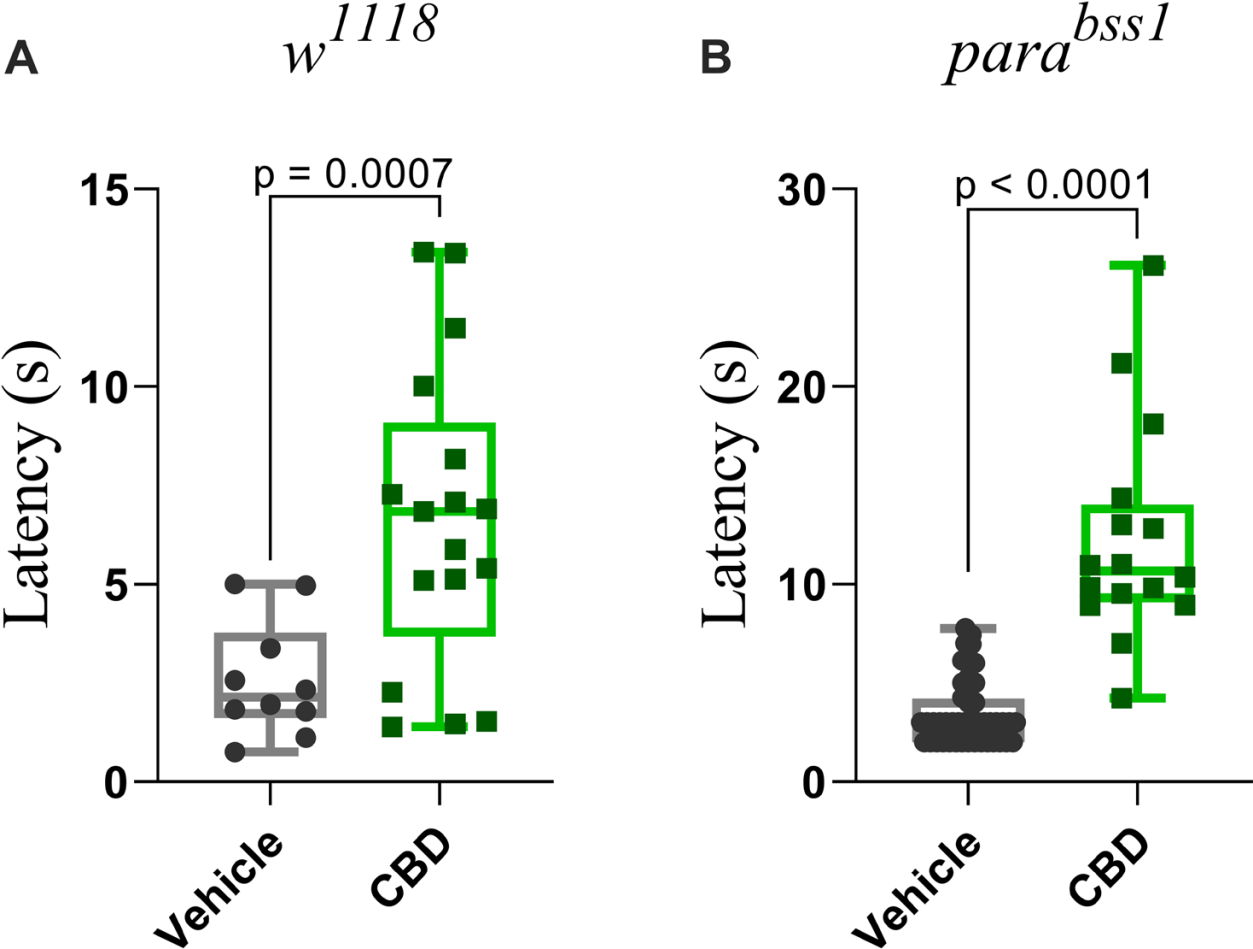
Cannabidiol (CBD) increases chemical nociception latency in both *w* ^1118^ and parabss1 larvae. (A) *w* ^*1118*^ third‐instar larvae treated with CBD for 1 h showed a significant increase in latency of nociceptive responses compared to vehicle controls (*p* = 0.0007; unpaired t‐test). (B) *para^bss1^* larvae treated with CBD showed a robust increase in response latency (*p* < 0.0001; Mann Whitney test). Each box represents the interquartile range, with the horizontal line indicating the mean. The whiskers represent the minimum and maximum values, respectively. Data are presented as individual data points (*n* ≥ 10 larvae per group).

Finally, to explore whether cannabidiol (CBD) could attenuate chemical nociception in flies, we tested both genotypes under CBD exposure. After 1 h of treatment, both *w* ^*1118*^ and *para* ^*bss1*^ larvae treated with CBD increased the latency time compared to vehicle. In *w* ^*1118*^ larvae, CBD treatment significantly increased the nociceptive latency compared to the vehicle group (*p* = 0.0007 unpaired *t*‐test), with an average latency of 6.63 ± 3.83 s versus 2.57 ± 1.47 s in the control group. This represents a 2.58‐fold increase, or a 158% increase in latency time. Similarly, *para* ^*bss1*^ larvae response increased in latency (*p* < 0.0001 Mann Whitney test), from 3.46 ± 1.67 s in the vehicle group to 12.26 ± 5.49 s in the CBD group, equivalent to a 3.54‐fold increase and a 254% increase (Figure 5A,B).

## 4 Discussion

The study of pain in animals raises ethical, philosophical and technical challenges. Ethically because the animal must be tested awake and is subject to discomfort; philosophically, pains as a subjective experience cannot be fully captured in non‐human organisms, given that its emotional dimension is uniquely human. In animal models, we can only evaluate the nociceptive response (Le Bars et al. 2001), the neural process that detects and reacts to potentially harmful stimuli. Some reviews have discussed the large number of animals used as models for chronic pain (Coderre and Laferrière 2020; Mogil 2009), mainly focusing on mammalian systems. Few reviews have addressed invertebrate models (Abboud et al. 2021; Burrell 2017).

In this study, we propose the *para* ^*bss1*^ mutant as a promising genetic model for investigating nociceptive sensitization in *Drosophila melanogaster* . A comparative analysis of human sodium channels (Na_v_1.7, Na_v_1.8 and Na_v_1.9) and the *para* gene in flies revealed a high degree of homology with Na_v_1.7, which is strongly associated with human pain syndromes (Cox et al. 2006). Three‐dimensional structural modelling further demonstrated that the human Na_v_1.7 R1150W mutation induces conformational changes, while no such structural alteration was observed in *para* ^*bss1*^, suggesting that its gain of function mechanism occurs through altered channel dynamics rather than major folding changes. Although our behaviour assays addressed acute responses, the persistent hyperexcitability of *para* ^*bss1*^ closely parallels mechanisms underlying chronic nociception. Behaviorally, *para* ^*bss1*^ larvae exhibited increased sensitivity to chemical stimuli but not thermal ones, compared to *w* ^*1118*^. When treated with CBZ, a sodium‐channel blocker, *w* ^*1118*^ had an increased nociceptive latency after 1 and 6 h of treatment with both tested concentrations, and para^bsss1^ larvae displayed a biphasic response: a mild increase in latency after 1 h at 3 mg/mL but not at 6 mg/mL, followed by the opposite pattern after 6 h, when only the higher dose produced a significant effect. This temporal inversion likely reflects the altered kinetics of sodium‐channel inactivation previously described for *para* ^*bss1*^ (Parker et al. 2011). Similarly, CBD significantly prolonged latency in both strains, supporting its analgesic potential and further validating the model for the pharmacological screening of candidate analgesics.

*Drosophila melanogaster* is a well characterised model for nociception studies. The anatomical and physiological aspects of class I‐IV neurons are well described (Karim and Moore 2011) and these neurons are responsible for mediating nociception in larvae (Cheng et al. 2010; Xiang et al. 2010; Yan et al. 2013). The first gene related to nociception in *D. melanogaster* was named *painless* and characterised in 2003, which encodes a non‐selective cation channel in the TRPA family that is responsible for thermal and mechanical nociception (Tracey et al. 2003). Its human orthologue, TRPA1, is likewise a key pain mediator (Souza Monteiro De Araujo et al. 2020).

Another important class of channels related to pain is the Na^+^ channel family, and to our knowledge, there is no previous information about the modulation of nociception by Na^+^ channels in *D. melanogaster* , supporting the novelty of our findings.

*Drosophila melanogaster* has two genes encoding Na^+^ channels (Na_v_1 and Na_v_2) in its genome, each in single copy (Liebeskind et al. 2011). There is little information in the literature about Na_v_2 in invertebrates. Thus, Na_v_1 is the predominant sodium channel gene in invertebrates (*para* gene in *D. melanogaster* ), and the orthologous Na_v_ channel in vertebrates is Na_v_1 (Zakon 2012).

In the human genome, 9 different genes encode Na_v_1 channels. The genes SCN1A to SCNA5A encode Na_v_1 to Na_v_1.5, and the genes SCN8A to SCN11A encode Na_v_1.6 to Na_v_1.19, respectively (Catterall 2014). Of particular interest are Na_v_1.7 (SCN9A), Na_v_1.8 (SCN10A), and Na_v_1.9 (SCN11A), which are predominantly expressed in peripheral sensory neurons and are directly associated with inherited human pain syndromes. Gain‐of‐function mutations in Na_v_1.7 have been implicated in inherited erythromelalgia, causing severe burning pain in the extremities (Dib‐Hajj et al. 2005). Similarly, mutations in Na_v_1.8 have been identified in patients with painful neuropathy and shown to increase sensory neuron excitability (Faber et al. 2012). Na_v_1.9 mutations have been linked to familial episodic pain syndromes characterised by recurrent episodes of intense pain (Halbritter et al. 2013). The human Na_v_1.7, Na_v_1.8 and Na_v_1.9 proteins were analysed in this work for comparison with *para* in *D. melanogaster* because variants in these genes have been associated with human Mendelian pain disease (Bennett et al. 2019).

In our work, we found that the molecular 3D models proposed in this work show no difference between protein from wild type allele (*para*) compared to mutant *para* ^*bss1*^. We found similar results for human mutant Na_V_ 1.7 (L858F) compared to human wild type Na_V_ 1.7, though the same mutational change of L to F the position on the protein is different. This human mutation (dbSNP rs80356476) has an autosomal dominant pattern of inheritance which is a gain of function and it promotes a hyperpolarizing shift in channel activation and an increase in the amplitude of the response to slow, small depolarizations (Han et al. 2006) similar to *para* ^*bss1*^ on electrophysiology (Parker et al. 2011) and 3D modelling.

The human mutant Na_V_ 1.7 (R1150W) is encoded by the *SCN9A* gene with mutation (dbSNP rs6746030) and it has an autosomal dominant pattern of inheritance with gain of function. This analysis was included in the present work due to its high frequency in the population (5.24% to 15.17%) according to PHARMAGKB (https://www.pharmgkb.org/, accessed in May 2021). This variation promotes a problem in the inactivation of Na_v_ 1.7v(R1150W) compared to the wild type and its mutation is related to more pain sensitivity in osteoarthritis, postamputation and sciatica (Reimann et al. 2010). Here, we demonstrate that the mutation R1150W in Na_v_1.7 3D provokes a conformational change in the 3D model structure, and it could be related to the increase in pain sensitivity.

After predicting the 3D protein structures, we validate the reproducibility of the chemical nociception assay using *w* ^*1118*^, the reference strain most widely used in *D. melanogaster* research (Ferreiro et al. 2018). The responses of third instar larvae exposed to HCl solution were consistent with those previously reported (Lopez‐Bellido et al. 2019). However, responses of *para* ^*bss1*^ larvae displayed markedly enhanced sensitivity to the same chemical stimulus compared to *w* ^*1118*^, which carries the wild type of *para* allele. This hypersensitive phenotype is consistent with the well‐characterised gain of function mutation in the *para* sodium channel (L1699F) (Parker et al. 2011). The *para* ^*bss1*^ mutation (L1699F) was originally characterised as a gain of function variant that enhances neuronal excitability and lowers the seizure threshold (Kroll et al. 2015; Loughney et al. 1989). Parker (Parker et al. 2011) demonstrated that flies carrying the Na_V_1–1L1699F transgene exhibited increased recovery time (from 38 to 49 s) and reduced seizure threshold (from 19.1 to 12.1 V) compared with controls, confirming that the L1699F substitution drives the hyperexcitable *para* ^*bss*1^ phenotype.

It is important to note that, although the *para* ^*bss1*^ mutation is best known for producing seizure‐like activity and bang‐sensitive paralysis in adult flies (Parker et al. 2011; Song and Tanouye 2006), such phenotypes have not been observed in larvae under standard laboratory conditions. In our assays, *para* ^*bss1*^ larvae exhibited normal locomotion prior to stimulation and consistently performed the characteristic nocifensive rolling behaviour in response to chemical stimuli, a rapid, coordinated 360° rotation mediated by class IV multidendritic nociceptors (Babcock et al. 2009; Tracey et al. 2003). This rolling response is highly stereotyped (Boivin et al. 2023; Sulkowski et al. 2011) and distinct from the spasmodic convulsions and transient paralysis reported in adult bang‐sensitive mutants, indicating that the behavioural responses described here reflect nociceptive activation rather than seizure activity.

The resulting neuronal hyperexcitability likely underlies the increased responsiveness of *para* ^*bss1*^ larvae to noxious chemical stimuli. Although the *para* ^*bss1*^ mutation is located at a different site from human Na_v_1.7 variants (Cox et al. 2006; Dib‐Hajj et al. 2005; Faber et al. 2012), these mutations share a common outcome: increased sodium channel activity and heightened sensory excitability. The *para* gene, orthologous to mammalian *SCN1A–SCN9A* genes, has been implicated in both seizure and nociceptive pathways, reinforcing its relevance as a model for sodium channel‐driven sensory dysfunction. Our approach thus represents a mutant‐based, rather than humanised model, that captures the shared pathophysiological principle of ion‐channel hyperexcitability. Future genetic engineering targeting residues homologous to Na_v_1.7 L858 or R1150 could further refine this system into a more precise model of human pain‐related sodium channelopathies.

Carbamazepine (CBZ) is a Na^+^ channel blocker (Kennebäck et al. 1995) widely used in the treatment of epilepsy (Beydoun et al. 2020) and chronic pain (Szok et al. 2019). As expected, CBZ modulated nociception in the *w* ^*1118*^ strain in 1 h of treatment. However, CBZ was already tested in the *para* ^*bss1*^ strain at the concentrations of 0.002–0.1 mg/mL and showed no results in altering the seizure phenotype (Reynolds et al. 2004), and therefore, *para* ^*bss1*^ was treated as a refractory treatment strain. Because of this, we tested doses higher than those previously reported in a short time (1 and 6 h) to avoid toxicity. The *para* ^*bss1*^ larvae responded to the lower concentration after 1 h of treatment, and after 6 h, only the highest concentration promoted some significant effect in increasing the latency, however, the delayed and dose‐dependent response to carbamazepine in *para* ^*bss1*^ does not undermine the model's validity; rather, it mirrors the pharmacoresistance observed in human sodium‐channel gain‐of‐function disorders such as SCN9A‐linked erythromelalgia and neuropathic pain (Baker and Nassar 2020; Dib‐Hajj et al. 2013; Fischer et al. 2009). We propose the use of this compound as a positive control in experiments with *para* ^*bss1*^ strain as a chronic nociception model for screening of new drugs.

Cannabidiol (CBD) has recently gained significant attention in pain management, leading to a growing body of research focused on cannabis‐based medical treatments. It is known that CBD promotes inactivation of the Na_v_1.7 channels (human) (Huang et al. 2023). Oral administration of CBD oil significantly increased response latency in both *D. melanogaster* models evaluated, indicating a reduction in nociceptive sensitivity. These findings suggest that CBD may modulate nociceptive pathways involving *para* channels, supporting the suitability and efficacy of the fruit fly models employed in this study for investigating CBD‐mediated pain mechanisms.

Although *Drosophila melanogaster* lacks clear orthologs of the canonical cannabinoid receptors CB1 and CB2 found in mammals, several studies suggest that phyto cannabinoids such as cannabidiol (CBD) can modulate neuronal activity through alternative mechanisms (Candib et al. 2024; Clarke et al. 2021). In the absence of classical endocannabinoid signalling, CBD may exert its effects by interacting with other molecular targets, including voltage‐gated sodium channels, transient receptor potential (TRP) channels, and regulators of oxidative or inflammatory homeostasis. Notably, *para* encodes the sole voltage‐gated sodium channel in *Drosophila*, which shares functional and pharmacological similarities with mammalian Na_v_ channels (BSC1A family) (Ravenscroft et al. 2023; Tapia et al. 2021). Thus, it is plausible that CBD's antinociceptive effects in *para* ^*bss1*^ mutants arise from its capacity to modulate sodium channel activity, either directly or indirectly, rather than through canonical cannabinoid receptors pathways. Consistent with this view, endocannabinoid‐related compounds have been reported to influence sodium channel function and excitability in *Drosophila* and other models (Jacobs and Sehgal 2020), supporting a noncanonical route through which CBD may regulate nociceptive responses.

In this work, we provide functional and pharmacological evidence supporting the use of the *D. melanogaster para* ^*bss1*^ mutant as a model for chronic nociception. Building on previous electrophysiological characterizations of the para channel (Parker et al. 2011), our results expand its application beyond seizure susceptibility, demonstrating that the same gain of function mutation confers enhanced chemical nociceptive sensitivity. The increased responsiveness to noxious stimuli, combined with the delayed effect of carbamazepine and the modulation by cannabidiol, highlight the translational potential of this model for exploring sodium channel related pain mechanisms.

Despite the relevance of these findings, this study has limitations. Our comparative and structural analyses revealed a strong sequence and domain conservation between para and human Na_v_1.7. However, we acknowledge that functional electrophysiological validation was not performed in this study. Demonstrating that human Na_v_1.7 can rescue para mutant phenotypes would indeed provide direct evidence of functional equivalence. Future work incorporating electrophysiological and genetic rescue approaches will be critical to further validate para mutants as translational models for human pain‐related sodium channelopathies.

Second, the focus on a single nociceptive assay may limit the generalizability of the model to other pain modalities. The effects of CBD are promising; however, the mechanism by which it modulates the para channel in flies requires further investigation. Although our study focused on the behavioural manifestations of para hyperactivity in nociceptive contexts, employing targeted RNAi in pan‐neuronal or sensory‐neuron drivers (e.g., elav‐Gal4, ppk‐Gal4) will be instrumental in refining circuit‐specific contributions to nociceptive hypersensitivity in *para* ^*bss1*^ larvae. Finally, even though *para* ^*bss1*^ serves as an effective model for gain‐of‐function channelopathy, future studies employing site‐directed mutagenesis (e.g., via CRISPR/Cas9) to create mutations in *Drosophila* that directly correspond to residues associated with human pain, such as L858F and R1150W in Na_v_1.7, would represent a powerful next step in creating even more accurate, translatable models of specific human pain syndromes.

In conclusion, our data supports the use of *para* ^*bss1*^ as a genetic model for chronic nociception in *D. melanogaster* . The model is sensitive to chemical stimuli, resistant to conventional sodium channel blockers and responsive to cannabidiol, offering a robust in vivo platform for screening novel analgesics targeting sodium channel dysfunction.

## Author Contributions

S.M.M. and L.I.V.C. Conception, design, development of methodology, analysis and interpretation of data, and writing of the manuscript. A.S.M. Experimental execution and data analysis. L.M.M.B. Manuscript writing. A.P.M.‐S. and J.G.H. provided support in writing and manuscript revision. F.S.E. Manuscript revision, technical and material support. C.U.‐V. Conception and design of the study, supervision, writing and revision of the manuscript, technical and material support.

## Funding

F.S.E. is financially supported by the Foundation for Research Support of the State of Minas Gerais (FAPEMIG, Grants APQ‐02185‐24 and APQ‐05981‐24). A.P.M.‐S. is supported by the Department of Psychiatry, College of Medicine, at the University of Saskatchewan. C.U.‐V is financially supported by the Foundation for Research Support of the State of Minas Gerais (FAPEMIG, Grants APQ 04689‐22, APQ‐02766‐17 and APQ‐00269‐22) and the National Council of Scientific and Technological Development (CNPq, grant number: 403193/2022‐2), FAPEMIG (grant number: CBB‐APQ‐03613‐17) for INCT‐TeraNano.

## Conflicts of Interest

The authors declare no conflicts of interest.
